# Focus on Clinical and Genetic Aspects of PKAN Through the Description of New Patients

**DOI:** 10.3390/genes16091008

**Published:** 2025-08-26

**Authors:** Marika Giuliano, Eugenia Borgione, Mariangela Lo Giudice, Francesco Domenico Di Blasi, Sandro Santa Paola, Girolamo Aurelio Vitello, Maurizio Elia, Roberto Russo, Corrado Romano, Carmela Scuderi

**Affiliations:** 1 Oasi Research Institute-IRCCS, Via Conte Ruggero 73, 94018 Troina, Italy; mgiuliano@oasi.en.it (M.G.); eborgione@oasi.en.it (E.B.); mlogiudice@oasi.en.it (M.L.G.); ssantapaola@oasi.en.it (S.S.P.); avitello@oasi.en.it (G.A.V.); melia@oasi.en.it (M.E.); rrusso@oasi.en.it (R.R.); cromano@oasi.en.it (C.R.); cscuderi@oasi.en.it (C.S.); 2 Department of Medicine and Surgery, Kore University of Enna, 94100 Enna, Italy; 3 Section of Clinical Biochemistry and Medical Genetics, Department of Biomedical and Biotechnological Sciences, University of Catania, 95123 Catania, Italy

**Keywords:** eye-of-the-tiger, *PANK2*, PKAN, NBIA

## Abstract

**Background/Objectives**: The most prevalent form of neurodegeneration with brain iron accumulation (NBIA) is pantothenate kinase-associated neurodegeneration (PKAN), caused by mutations in the *PANK2* gene. The hallmark of PKAN is the “eye-of-the-tiger” sign, which is characterized by a bilateral region of central hyperintense signal surrounded by a hypointense signal in the medial globus pallidus on T2-weighted brain magnetic resonance imaging (MRI). **Methods**: Whole-exome sequencing (WES) was performed in four patients who presented with dystonia, cognitive impairment and abnormalities of the globus pallidus. All patients underwent comprehensive clinical and instrumental evaluations. **Results**: Molecular analysis using WES revealed *PANK2* variants in all four cases. Two patients were homozygous for the known pathogenic variant c.1169A > T (p.N390I). The remaining two patients displayed compound heterozygotes, each carrying the novel splicing variant c.906-1G > A on one allele, combined with a different second variant on the other allele: the new missense variant c.617G > A (p.G206D) in one case and the known pathogenic variant c.1231G > A (p.G411R) in the other. In one case, brain imaging documented the transition from initial hyperintensity of the globus pallidus to the development of the “eye-of-the-tiger” sign. In two cases, MRI findings clearly demonstrated the characteristic “eye-of-the-tiger” appearance. Ultimately, in one case, the imaging likely captured a later disease stage, in which the “eye-of-the-tiger” sign was no longer visible, and only the residual hypointensity remained. **Conclusions**: This study describes two novel likely pathogenic variants and documents the full MRI progression of globus pallidus involvement in PKAN. The sequence starts with early T2 hyperintensity, followed by the emergence of the typical “eye-of-the-tiger” sign, and culminates in marked hypointensity in advanced stages. Since the initial clinical presentation may mimic mitochondrial disorders or other neurometabolic conditions, these imaging features are crucial for guiding differential diagnosis and enabling accurate disease identification.

## 1. Introduction

Neurodegeneration with brain iron accumulation (NBIA), characterized by iron accumulation in certain areas of the brain, mainly the globus pallidus and substantia nigra, constitutes a group of rare, progressive, inherited neurodegenerative diseases [1,2]. The age of onset of NBIA ranges from early childhood to late adulthood, and clinical features include slowly progressive pyramidal and extrapyramidal symptoms, spasticity, cognitive impairment, and neuropsychiatric disorders [1,3]. It is estimated that NBIA affects 15,000 to 20,000 individuals worldwide [1]. Depending on the mutated gene that causes the disorder and on magnetic resonance imaging (MRI), many types of NBIA can be differentiated [4].

The most prevalent form of NBIA is pantothenate kinase-associated neurodegeneration (PKAN), which accounts for approximately 35–50% of the NBIA patient population [5]. It is caused by mutations in the *PANK2* gene and is inherited in an autosomal recessive manner. The *PANK2* gene is located on chromosome 20 and the functional protein exists as a dimer. There are four genes in the human genome that encode protein isoforms of the Pank enzymes: *PANK1* (producing PANK1α and PANK1β), *PANK2*, *PANK3*, and *PANK4*. These isoforms play a crucial role in the biosynthesis of coenzyme A (CoA), sharing a conserved C-terminal domain but possessing distinct N-terminal regions [6,7]. PANK genes differ in their tissue distribution and localization. In particular, the *PANK2* gene is highly expressed in the brain and is localized in the mitochondrial intermembrane space [7].

Despite being rare, the most common NBIA disorder, PKAN is recognizable by its distinctive clinical and radiographic features [8]. The major symptoms of PKAN include progressive pyramidal and extrapyramidal signs, retinitis pigmentosa, optic atrophy, and dementia. The age at which the disease begins usually corresponds to how quickly it progresses; earlier onset is associated with faster progression, whereas later onset tends to follow a slower, more gradual course.

PKAN has been classified clinically as classic or atypical [3]. Classic PKAN manifests in the first decade of life with rapid disease progression, characterized by prominent dystonia and the loss of the ability to walk within 10 to 15 years of symptom onset. The most common early signs are gait abnormalities and clumsiness caused by lower limb dystonia. Many affected children also show early signs of developmental delay and dyspraxia before full-blown dystonia and spasticity appear. Retinitis pigmentosa usually progresses more slowly than motor dysfunction.

The atypical form appears later in life with a slower disease progression and a broader clinical spectrum. The loss of ability to walk usually occurs more gradually, over 15–40 years. Later onset tends to correlate with a parkinsonism-predominant presentation, while adolescent-onset cases may exhibit a mix of dystonia and parkinsonism. Early symptoms may include neuropsychiatric features and speech disturbances.

Several mutations in the PKAN gene have been reported in the literature, mainly nonsense and missense variants associated with the typical and atypical forms, respectively [9].

The hallmark of this disease is the “eye-of-the-tiger” sign, which is characterized by a bilateral area of central hyperintense signal surrounded by a hypointense signal in the medial globus pallidus on T2-weighted brain MRI, reflecting focal accumulation of iron in this region [10]. This imaging pattern is strongly associated with PKAN; however, exceptions have been documented. These include cases in which other conditions produce a similar MRI appearance, as well as individuals with a confirmed clinical and genetic diagnosis of PKAN who do not exhibit this characteristic sign. Notably, the “eye-of-the-tiger” sign has been previously reported in other forms of NBIA, such as Mitochondrial membrane protein-associated neurodegeneration (MPAN) [11] and Coenzyme A synthase protein-associated neurodegeneration (CoPAN) [12], and cases with Wilson’s disease [13,14]. However, according to most imaging findings reported in the literature, the “eye-of-the-tiger” sign is absent in some patients with PKAN [15,16], whereas in others, the signal—initially present—disappeared over time [17].

Therefore, careful differential diagnosis is essential to ensure an accurate final assessment.

Here, we report detailed clinical and genetic findings of four unrelated cases with PKAN. In particular, we described two new probably pathogenic variants (c.906–1G > A and c.617G > A (p.G206D)), and documented the full MRI progression of globus pallidus involvement in this disorder.

## 2. Materials and Methods

### 2.1. Patient Examination

The patients were referred to our Institute for a comprehensive diagnostic evaluation aimed at investigating the underlying condition. A thorough multidisciplinary clinical assessment was performed for all patients, including neurological and physical examination, as well as cognitive profiling. Instrumental investigations included brain MRI, electroencephalography (EEG), and electromyography (EMG).

### 2.2. Whole-Exome Sequencing (WES)

After obtaining informed consent, the genomic DNA was extracted from patients’ peripheral blood using standard protocols. WES was subsequently performed to investigate the genetic etiology of the condition.

Molecular testing was conducted using WES on the NextSeq 550 System platform (Illumina, San Diego, CA, USA) following the manufacturer’s protocols, with raw sequencing reads aligned to the human reference genome (GRCh37/hg19). The resulting binary alignment map (BAM) files were examined with Integrative Genomics Viewer (IGV) software (v2.5.3). Variant calling was conducted using the Genome Analysis Toolkit (GATK) pipeline (https://gatk.broadinstitute.org/hc/en-us/ (accessed on 3 May 2025)), and functional annotation of variants was performed using ANNOVAR (http://wannovar.wglab.org). Only rare variants were considered; those with a minor allele frequency (MAF) greater than 1% were excluded based on data from 1000 Genome Project (http://1000genomes.org/ (accessed on 20 June 2025)), Exome Aggregation Consortium (http://exac.broadinstitute.org/ (accessed on 20 June 2025)), and Genome Aggregation Database (https://gnomad.broadinstitute.org/ (accessed on 20 June 2025)). Additionally, variants annotated by ClinVar (https://www.ncbi.nlm.nih.gov/clinvar/ (accessed on 20 June 2025)) as benign or likely benign were filtered out, as well as synonymous variants without predicted impact on splicing. The remaining variants were evaluated and categorized according to the American College of Medical Genetics and Genomics (ACMG) into variants of uncertain significance (VUS), likely pathogenic, or pathogenic. The potential pathogenicity of single nucleotide variants was assessed using in silico tools including SIFT (http://sift.bii.a-star.edu.sg/ (accessed on 20 June 2025)), PolyPhen-2 (http://genetics.bwh.harvard.edu/pph2/ (accessed on 20 June 2025)), Mutation Taster (http://www.mutationtaster.org/ (accessed on 20 June 2025)) and, specifically for splice site variants, SpliceAI (https://spliceailookup.broadinstitute.org/ (accessed on 20 June 2025)).

Confirmation and segregation analyses of the putative pathogenic variant were performed by Sanger sequencing on the probands’ and parents’ DNA. PCR primers of the *PANK2* gene were designed using the software Vector NTI Advance 10.3.0 (Informax, Frederick, MD, USA). The reference sequences used were the *PANK2* gene (NM_001386393.1). PCR reactions were performed according to the manufacturer’s instructions. PCR products were sequenced bidirectionally using a BigDye Terminator Cycle Sequencing Kit. Products were purified with a DyeEx 2.0 Spin Kit (Qiagen, Hilden, Germany) and analyzed on an ABI310 automated DNA sequencer (Applied Biosystems, Foster City, CA, USA). Patient sequence data were aligned for comparison with the corresponding wild-type variant.

3D molecular modeling and structural analysis of both mutant and wild-type proteins were carried out using UCSF ChimeraX version 1.9.

### 2.3. cDNA Analysis

Total RNA was extracted from peripheral blood leukocytes using the RNeasy Mini kit (Qiagen, Hilden, Germany), following manufacturer’s protocol. To minimize genomic DNA contamination prior to reverse transcription, the RNA samples were incubated at 42 °C with the specific Wipeout buffer provided in the QuantiTect Reverse Transcription kit (Qiagen, Hilden, Germany). Subsequently, 600 ng of total RNA was reverse transcribed into cDNA for PCR amplification using primers targeting exons 3 (forward: 5′-GCAAACTGGATGAACTAGATTGC-3′) and 5 (reverse: 5′-GCACACATTCTTGCTATTGAGC-3′).

## 3. Results

### 3.1. Patient’s Clinical History

**Figure 1:**
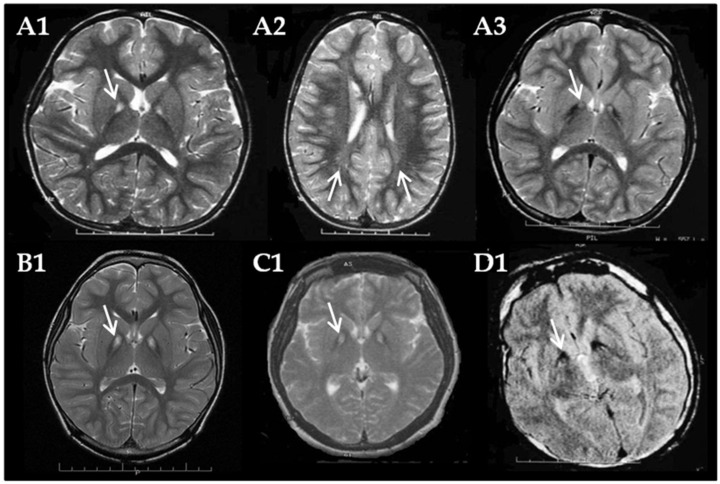
Full MRI progression of globus pallidus involvement in four patients with PKAN (**A1**–**D1**). (**A1**–**A3**) Imaging of case 1. At age of 3 years, bilateral hyperintensity of the globus pallidus (white arrow) (**A1**) and abnormal periventricular white matter signal (white arrows) (**A2**) are observed. At age 9, the characteristic “eye-of-the-tiger” sign is evident (white arrow) (**A3**). (**B1**) Case 2 showing the “eye-of-the-tiger” sign (white arrow). (**C1**) Case 3 with the “eye-of-the-tiger” sign (white arrow). (**D1**) Case 4 showing bilateral hypointensity of the globus pallidus (white arrow).

Case 1 was a 13-year-old boy, born at term by cesarean delivery after a physiological pregnancy, to non-consanguineous parents. The paternal cousin has intellectual disability (ID) and difficulty walking. At 16 months, he started walking with an unsteady gait. Speech and language were delayed. At the age of 3 years, examination showed global developmental delay, dysmorphisms, dysarthric language, spastic paraparesis, and diffuse muscular wasting. Brain MRI showed bilateral hyperintensity of the globus pallidus (Figure 1(A1)) and periventricular white matter abnormal signals (Figure 1(A2)). At the age of 9, he presented with mild ID, retinitis pigmentosa, bilateral conductive hearing loss, and persistent diffuse dystonic movements. He also lost the ability to walk at that time. EMG showed myogenic signs. Brain MRI showed the “eye-of-the-tiger” sign (Figure 1(A3)). At the last clinical examination, the patient exhibited continuous and diffuse dystonic movements, self-inflicted injuries to the lower lip, cheeks, and tongue, necessitating the use of thermo-printed dental plates.

Case 2, a 7-year-old boy, was born to non-consanguineous parents at term by physiological delivery, after maternal abnormal weight gain during pregnancy. A first cousin presented global developmental delay, and a first cousin of his father had ID and behavioral disturbances. He had normal psychomotor development and autonomous ambulation at 13 months with frequent falls. He presented language delay. At the age of 4 years, he had ataxia and global developmental delay. At the age of 6 years, the patient presented with mild ID, dystonia, gait worsening, elevation in creatine kinase levels (223 U/L; *n.v*. 24–204 U/L), and bilateral Babinski sign. Brain MRI revealed the presence of symmetrical signal abnormalities in the globus pallidus exhibiting the characteristic “eye-of-the-tiger” sign (Figure 1(B1)).

Case 3 was a 33-year-old woman born to non-consanguineous parents after physiological pregnancy and delivery. A sister presented ID. After initial normal psychomotor development, at the age of 3 years, she presented with hyperactivity, speech disorders, dysphagia, paresthesias, muscle cramps and progressive gait difficulties until losing ambulation. At age 12, the presence of retinitis pigmentosa was highlighted. At the age 20, she presented with mild ID and ataxia. At the age of 29 years, the patient had three episodes characterized by loss of consciousness and falls, upper limb clonus, and lower limb hypertonia for a few minutes.

On examination, dysarthria, resting tremor with dystonic movements of her hands, moderate muscle hypertonia and hypertrophy in the lower limbs, ataxic gait and retinitis pigmentosa were observed. EEG showed multifocal spikes. EMG showed neurogenic signs. Brain MRI showed symmetrical signal alterations of the globus pallidus on both sides consistent with the characteristic “eye-of-the-tiger” sign (Figure 1(C1)). At age 30, progressive worsening of gait, motor coordination, speech and dysphagia was reported, with the addition of frequent urination and constipation.

Case 4, a 38-year-old man, was born from first-cousins’ parents after normal pregnancy and physiological delivery. Family history was negative. He had mild psychomotor delay, but he presented with toe walking by the age of 18 months and learning difficulties at school age. Dystonic movements and pyramidal signs appeared later and, at the age of 13 years, the patient lost the ability to walk. At examination, verbal language was absent, dementia, spastic tetraparesis, dystonic movements, diffuse muscle atrophy and retinitis pigmentosa were present. Creatine kinase was slightly elevated (340 U/L; *n.v.* 24–204 U/L). EMG revealed severe demyelinating sensorimotor neuropathy. Brain MRI showed bilateral hypointensity of the globus pallidus (Figure 1(D1)).

### 3.2. Genetic and Functional Analysis

Cases 1 and 4 were homozygous for the known pathogenic variant c.1169A > T (p.N390I) in the *PANK2* gene. This variant was absent from both the ExAC and 1000 Genomes Project databases, and it had a frequency of 0.000004 in gnomAD. It is considered likely pathogenic according to the variant interpretation guidelines of ACMG (criteria PM1, PP2, PM2, PP3, PP5) (Table 1), and is predicted to be probably damaging by PolyPhen-2 (score = 1), deleterious by SIFT (score = 0), and disease-causing by Mutation Taster (score = 1). This variant was first described by Zhou et al. (2001) [18] in a patient with a classical form of PKAN.

Case 2 was a compound heterozygous for two variants: the known pathogenic missense variant c.1231G > A (p.G411R), inherited from his mother, and the novel variant c.906-1G > A on the splice acceptor site, inherited from his father. The variant c.1231G > A (p.G411R) was absent from the 1000 Genomes Project databases, and it had a frequency of 0.0001 in ExAC and 0.00013 in gnomAD. It is considered pathogenic according to ACMG guidelines (criteria PM3, PP1, PS3, PP3, PM2, PP2) (Table 2), and is also predicted to be probably damaging by PolyPhen–2 (score = 1), deleterious by SIFT (score = 0), and disease-causing by Mutation Taster (score = 1). This variant has also been reported by Zhou et al. (2001) [18] in patients with both classical and atypical forms of PKAN. The second variant, c.906-1G > A, was absent from the ExAC, 1000 Genomes Project databases, and gnomAD. It is considered pathogenic according to ACMG guidelines (criteria PM3, PVS1, PM2) (Table 3), is predicted to be disease-causing by Mutation Taster (score = 1), and SpliceAI indicates that it may alter splicing (acceptor gain score 0.71, acceptor loss score 0.99). The nucleotide sequence of the splice acceptor region is highly conserved across species, as shown by Multiz 100-species alignment analysis (UCSC Genome Browser, http://genome.ucsc.edu/ (accessed on 13 August 2025).

Case 3 was compound heterozygous for two novel variants in the *PANK2* gene: the c.617G > A (p.G206D), inherited from her mother, and the c.906-1G > A, also described in case 2, for which paternal inheritance could not be confirmed. The first variant, c.617G > A (p.G206D), was absent from the ExAC and 1000 Genomes Project databases, and it had a frequency of 0.000004 in gnomAD. It is considered likely pathogenic according to ACMG guidelines (criteria PM1, PP2, PM2, PP3) (Table 4), and it also predicted to be probably damaging by PolyPhen-2 (score = 1), deleterious by SIFT (score = 0), and disease-causing by Mutation Taster (score = 1). The amino acid Gly206 is highly conserved among vertebrates according to Multiz 100-species alignment analysis, suggesting a potential functional relevance.

**Figure 2:**
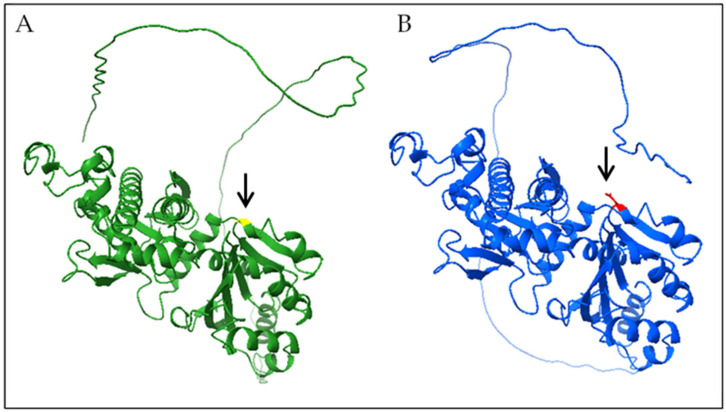
Three-dimensional modeling of the wild-type (green) and mutant (blue) protein structures showed an anomalous conformation and, respectively, Gly206 (yellow) and Asp206 (red) were highlighted.

We carried out 3D modeling of mutant and wild-type protein structures (Figure 2), using UCSF ChimeraX version 1.9, and observed an abnormal conformational change.

**Figure 3:**
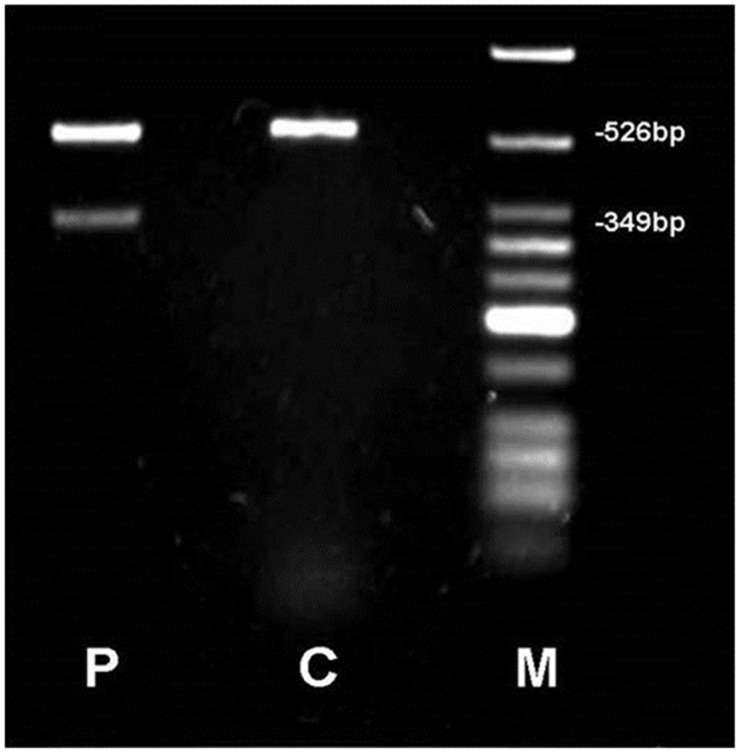
cDNA analysis of the c.906–1G > A. RT-PCR products were resolved by agarose gel electrophoresis. P = patient, C = control, M = molecular marker. Wild-type genotype corresponds to a 526bp fragment; the splice-site variant leads to an aberrant band of 349bp.

The second variant, c.906-1G > A, leads to an abnormal splicing event affecting the splice acceptor site of intron 3, resulting in the skipping of exon 4, which was confirmed by cDNA analysis (Figure 3). We investigated its impact on the transcript coding sequence, using PCR amplification of cDNA in the case 3 and in normal control producing distinct patterns. Specifically, the control sample produced a single 526 bp band, whereas the case cDNA sample showed two bands: the normal 526 bp band and an additional aberrant 349 bp band. This finding confirms exon 4 skipping and suggests the generation of an abnormal protein product.

## 4. Discussion

In this study, we present four patients with variants in the *PANK2* gene. All our patients had dystonia and three out of four had ID, while the other had dementia. Additional signs were retinitis pigmentosa, ataxia and spastic paraplegia (Table 5). Although the initial clinical presentation of case 1 resembled a mitochondrial disorder, and case 4 appeared similar to other NBIA conditions, a complete diagnostic evaluation over time and molecular analysis through WES revealed four PKAN patients. In particular, two patients were homozygous for the known pathogenic variant c.1169A > T (p.N390I), while the other two were compound heterozygotes, both carrying the novel splicing variant c.906-1G > A on one allele. On the second allele, one patient had the known pathogenic variant c.1231G > A (p.G411R) and the other carried the novel missense variant c.617G > A (p.G206D).

It is interesting to note that cases 1 and 4, who share the same homozygous variant, come from the same small town in the province of Agrigento (Sicily), with fewer than 9000 inhabitants. This finding leads us to hypothesize a founder effect in that geographic area.

The splicing variant c.906-1G > A, found in cases 2 and 3, is described for the first time in this study. PCR of the cDNA derived from the patient 3 showed two bands, one normal and one aberrant confirming the skipping of exon 4 (Figure 3). It is expected to lead to either the absence or truncation of the protein product and/or nonsense-mediated mRNA decay. Unfortunately, it was not possible to extract cDNA from patient 2 to perform a replicate experiment and also isolate the bands for validation by sequencing. This represents a limitation of the study. Validating these results in additional, unrelated cases carrying the same variant strengthen the evidence supporting its pathogenicity classification in future research.

Also, the variant c.617G > A (p.G206D), is reported for the first time in this study. In silico predictive software suggests a possible pathogenic role. The Gly206 residue is highly conserved across species, indicating a critical role in maintaining the structural integrity and enzymatic function of *PANK2*. Substitution of this small uncharged glycine with a negatively charged aspartic acid, as shown by 3D modeling, likely disrupts the local protein conformation due to steric obstruction and altered electrostatic interactions. These structural perturbations may impair substrate binding or catalytic activity of *PANK2*. The pathogenic role of the c.617G > A (p.G206D) variant has been investigated primarily through in silico predictions and 3D modeling. However, the absence of cell-based or biochemical functional studies represents a limitation of this study, which could be addressed in future research.

*PANK2* is localized in mitochondria and catalyze the initial step in the synthesis of CoA, an essential molecule for energy metabolism and lipid synthesis and degradation. The deficiency of phosphopantotenate, a product of the reaction catalyzed by *PANK2*, would result in high local cysteine levels, a potent iron chelator, leading to secondary iron accumulation in the brain and secondary oxidative stress-induced neuronal injury [19].

Iron plays a crucial role in the correct metabolism of brain cells, being a key factor in multiple processes such as: energy production, DNA synthesis and repair, phospholipid metabolism, myelin synthesis and production of neurotransmitters [20]. Brain iron deficiency may result in cognitive impairment and neuropsychiatric disorders [20]. Conversely, excessive iron accumulation in the brain contributes to the progression of neurological conditions, including Alzheimer’s disease, Parkinson’s disease, and stroke [20]. The free iron is highly reactive and can be destructive to the cells and induce cell death.

In fact, in the typical “eye-of-the-tiger” sign from T2-weighted MRI, the central hyperintense signal probably occurs due to the dramatic rarefaction of the tissue and the microglial activation, whereas the area of reduced signal is due to iron accumulation in the tissue. Finally, the advanced phase of the disease is characterized by the presence of hypointensity alone. Over time, severe gliosis and iron deposit accumulation likely reduce signal intensity, causing the disappearance of “eye-of-the-tiger” sign.

In this study, we documented, in four patients, the full MRI progression of globus pallidus involvement in PKAN. In the first case, the brain MRI initially revealed the hyperintensity of the globus pallidus and periventricular white matter abnormalities, neuroradiological features frequently observed in mitochondrial disorders. Approximately six years after, the “eye-of-the-tiger” sign became noticeable. The clearly “eye-of-the-tiger” sign was immediately identified on brain MRI in case 2 and 3. In the oldest patient, case 4, presenting the same variant observed in case 1, the imaging likely captured a later stage, characterized by only bilateral residual hypointensity of the globus pallidus, neuroradiological features commonly observed in other NBIA forms. It is likely that the delay in the patient’s referral and subsequent MRI contributed to this.

It has been reported that the sign was initially present but disappeared over time in PKAN patient [17]. Similarly, the unusual absence of this sign has also been described in the literature [21].

Whereas Delgrado et al. (2011) [21] reported no correlation between the presence of the “eye-of-the-tiger” sign and clinical data, age of onset, or gender, our patient cohort demonstrated a clear association between age and characteristic radiological finding and severity of the clinical phenotype. Specifically, hyperintensity of the globus pallidus was observed prior to the appearance of the “eye-of-the-tiger” sign, while residual hypointensity was more commonly seen after the hallmark sign had developed. Moreover, in our case 4, the oldest patient who presented with hypointensity of the globus pallidus, the phenotype was the most severe.

This relationship highlights the importance of considering age at onset in both clinical evaluation and research, supporting the utility of age-stratified analyses that potentially can provide earlier diagnosis and personalize management strategies.

This study, consistent with the existing literature, confirms that the “eye-of-the-tiger” sign, when present, is strongly associated with mutations in the *PANK2* gene. However, it also highlights that the timing of clinical observation may not always permit the visualization of this radiological feature typically observed in PKAN. Our findings indicate that the sign may be absent at certain stages of the disease or may instead present as nonspecific hypo- or hyperintense changes on MRI, in agreement with the literature. However, the observed MRI progression is based on patients at different ages and disease stages, rather than on serial scans of the same individuals. Only case 1 includes longitudinal imaging. Unfortunately, no earlier or follow-up MRIs are available for the other cases, limiting our ability to show the progression of globus pallidus involvement in this disorder.

In summary, we report four cases of patients with PKAN, including two novel likely pathogenic variants and the variability of the characteristic “eye-of-the-tiger” sign.

It is therefore essential to find the right moment when the “eye-of-the-tiger” sign is visible.

## Figures and Tables

**Table 1:** Variant c.1169A > T (p.N390I) classification according to the ACMG criteria.

c.1169A > T (p.N390I)		
Criteria for Classifying Variants	Category Code	Description
Pathogenic Moderate:	PM1	Non-truncating non-synonymous variant is located in a mutational hot spot and/or critical and well-established functional domain
Pathogenic Supporting:	PP2	Missense variant in a gene with low rate of benign missense mutations and for which missense mutation is a common mechanism of a disease
Pathogenic Moderate:	PM2	Extremely low frequency in gnomAD population databases
Pathogenic Moderate:	PP3	For a missense or a splicing region variant, computational prediction tools unanimously support a deleterious effect on the gene
Pathogenic Supporting:	PP5	Reputable source recently reports variant as pathogenic, but the evidence is not available to the laboratory to perform an independent evaluation
ACMG variant classification		Likely Pathogenic

**Table 2:** Variant c.1231G > A (p.G411R) classification according to the ACMG criteria.

c.1231G > A (p.G411R)		
Criteria for Classifying Variants	Category Code	Description
Pathogenic Very Strong:	PM3	For recessive disorders, detected in trans with a pathogenic variant, or in a homozygous or compound heterozygous state in affected cases
Pathogenic Supporting:	PP1	Cosegregation with disease in multiple affected family members in a gene definitively known to cause the disease
Pathogenic Supporting:	PS3	Well-established functional studies show damaging effect on the gene or gene product
Pathogenic Strong:	PP3	For a missense or a splicing region variant, computational prediction tools unanimously support a deleterious effect on the gene
Pathogenic Moderate:	PM2	Extremely low frequency in gnomAD population databases
Pathogenic Supporting:	PP2	Missense variant in a gene with low rate of benign missense mutations and for which missense mutation is a common mechanism of a disease
ACMG variant classification		Pathogenic

**Table 3:** Variant c.906-1G > A classification according to the ACMG criteria.

c.906-1G > A		
Criteria for Classifying Variants	Category Code	Description
Pathogenic Very Strong:	PM3	For recessive disorders, detected in trans with a pathogenic variant, or in a homozygous or compound heterozygous state in affected cases
Pathogenic Strong:	PVS1	Null variant in a gene where loss of function is a known mechanism of disease
Pathogenic Moderate:	PM2	Extremely low frequency in gnomAD population databases
ACMG variant classification		Pathogenic

**Table 4:** Variant c.617G > A (p.G206D) classification according to the ACMG criteria.

c.617G > A (p.G206D)		
Criteria for Classifying Variants	Category Code	Description
Pathogenic Moderate:	PM1	Non-truncating non-synonymous variant is located in a mutational hot spot and/or critical and well-established functional domain
Pathogenic Supporting:	PP2	Missense variant in a gene with low rate of benign missense mutations and for which missense mutation is a common mechanism of a disease
Pathogenic Moderate:	PM2	Extremely low frequency in gnomAD population databases
Pathogenic Strong:	PP3	For a missense or a splicing region variant, computational prediction tools unanimously support a deleterious effect on the gene
ACMG variant classification		Likely Pathogenic

**Table 5:** Clinical features in four patients with PKAN.

Case	Sex	Age/y	Age/mo of Onset	*PANK2* Variants	Cognitive Levels	Language Disorders	Spastic Paraplegia	Retinitis Pigmentosa	Deambulation	Dystonia	Imaging Features on MRI
1	M	13	16	c.1169A > T (p.N390I)	Mild ID	+	+	+	Non-ambulatory	+	At 3 years: bilateral hyperintensity of the globus pallidus and white matter abnormalities At 9 years: eye-of-the-tiger sign
2	M	7	13	c.1561G > A (p.G521R) c.906-1G > A	Mild ID	+	−	−	Ataxic gait	+	Eye-of-the-tiger sign
3	F	33	36	c.617G > A p.(G206D) c.906-1G > A	Mild ID	+	−	+	Ataxic gait	+	Eye-of-the-tiger sign
4	M	38	18	c.1169A > T (p.N390I)	Dementia	+	+	+	Non-ambulatory	+	Bilateral hypointensity of the globus pallidus

## Data Availability

Data are available from the corresponding author upon a reasonable request.

## Abbreviations

The following abbreviations are used in this manuscript:

NBIA	Neurodegeneration with brain iron accumulation
MRI	Magnetic resonance imaging
PKAN	Pantothenate kinase-associated neurodegeneration
CoA	Coenzyme A
MPAN	Mitochondrial membrane protein-associated neurodegeneration
CoPAN	Coenzyme A synthase protein-associated neurodegeneration
EEG	Electroencephalography
EMG	Electromyography
WES	Whole-exome sequencing
ID	Intellectual disability
ACMG	American College of Medical Genetics

## References

[1] Wydrych A., Pakuła B., Janikiewicz J., Dobosz A.M., Jakubek-Olszewska P., Skowrońska M., Kurkowska-Jastrzębska I., Cwyl M., Popielarz M., Pinton P. (2025). Metabolic Impairments in Neurodegeneration with Brain Iron Accumulation. Biochim. Biophys. Acta (BBA)-Bioenerg..

[2] Iankova V., Karin I., Klopstock T., Schneider S.A. (2021). Emerging Disease-Modifying Therapies in Neurodegeneration with Brain Iron Accumulation (NBIA) Disorders. Front. Neurol..

[3] Hajati R., Emamikhah M., Danaee Fard F., Rohani M., Alavi A. (2022). Neurodegeneration with Brain Iron Accumulation and a Brief Report of the Disease in Iran. Can. J. Neurol. Sci..

[4] Aoun M., Corsetto P.A., Nugue G., Montorfano G., Ciusani E., Crouzier D., Hogarth P., Gregory A., Hayflick S., Zorzi G. (2017). Changes in Red Blood Cell Membrane Lipid Composition: A New Perspective into the Pathogenesis of PKAN. Mol. Genet. Metab..

[5] Marupudi N., Xiong M.P. (2024). Genetic Targets and Applications of Iron Chelators for Neurodegeneration with Brain Iron Accumulation. ACS Bio Med. Chem. Au.

[6] Alfonso-Pecchio A., Garcia M., Leonardi R., Jackowski S. (2012). Compartmentalization of Mammalian Pantothenate Kinases. PLoS ONE.

[7] Kwinta R., Kopcik K., Koberling A. (2024). Pathology and Treatment Methods in Pantothenate Kinase-Associated Neurodegeneration. Postep. Psychiatr. Neurol..

[8] Gregory A., Hayflick S.J., Adam M.P., Feldman J., Mirzaa G.M., Pagon R.A., Wallace S.E., Amemiya A. (1993). Pantothenate Kinase-Associated Neurodegeneration. GeneReviews^®^.

[9] Chang X., Zhang J., Jiang Y., Wang J., Wu Y. (2020). Natural History and Genotype-Phenotype Correlation of Pantothenate Kinase-Associated Neurodegeneration. CNS Neurosci. Ther..

[10] Santambrogio P., Ripamonti M., Cozzi A., Raimondi M., Cavestro C., Di Meo I., Rubio A., Taverna S., Tiranti V., Levi S. (2022). Massive Iron Accumulation in PKAN-Derived Neurons and Astrocytes: Light on the Human Pathological Phenotype. Cell Death Dis..

[11] Dehghan Manshadi M., Rohani M., Rezaei A., Aryani O. (2022). A Case of MPAN with “Eye of the Tiger Sign,” Mimicking PKAN. Mov. Disord. Clin. Pr..

[12] Evers C., Seitz A., Assmann B., Opladen T., Karch S., Hinderhofer K., Granzow M., Paramasivam N., Eils R., Diessl N. (2017). Diagnosis of CoPAN by Whole Exome Sequencing: Waking up a Sleeping Tiger’s Eye. Am. J. Med. Genet. A.

[13] Litwin T., Karlinski M., Skowrońska M., Dziezyc K., Gołębiowski M., Członkowska A. (2014). MR Image Mimicking the “Eye of the Tiger” Sign in Wilson’s Disease. J. Neurol..

[14] Kumar S., Pandey S., Malhotra H.S., Garg R.K. (2024). Is the “Eye of Tiger” Really Emblematic of Pantothenate Kinase-Associated Neurodegeneration Type 1? An Uncommon MR Image in Wilson’s Disease. Neurol. India.

[15] Hayflick S., Westaway S. (2006). Pantothenate Kinase 2 Mutation without “eye-of-the-Tiger” Sign. Pediatr. Radiol..

[16] Zolkipli Z., Dahmoush H., Saunders D.E., Chong W.K.K., Surtees R. (2006). Pantothenate Kinase 2 Mutation with Classic Pantothenate-Kinase-Associated Neurodegeneration without “eye-of-the-Tiger” Sign on MRI in a Pair of Siblings. Pediatr. Radiol..

[17] Baumeister F.A.M., Auer D.P., Hörtnagel K., Freisinger P., Meitinger T. (2005). The Eye-of-the-Tiger Sign Is Not a Reliable Disease Marker for Hallervorden-Spatz Syndrome. Neuropediatrics.

[18] Zhou B., Westaway S.K., Levinson B., Johnson M.A., Gitschier J., Hayflick S.J. (2001). A Novel Pantothenate Kinase Gene (*PANK2*) Is Defective in Hallervorden-Spatz Syndrome. Nat. Genet..

[19] Kurian M.A., Hayflick S.J. (2013). Pantothenate Kinase-Associated Neurodegeneration (PKAN) and PLA2G6-Associated Neurodegeneration (PLAN): Review of Two Major Neurodegeneration with Brain Iron Accumulation (NBIA) Phenotypes. Int. Rev. Neurobiol..

[20] Gao G., You L., Zhang J., Chang Y.-Z., Yu P. (2023). Brain Iron Metabolism, Redox Balance and Neurological Diseases. Antioxidants.

[21] Delgado R.F., Sanchez P.R., Speckter H., Then E.P., Jimenez R., Oviedo J., Dellani P.R., Foerster B., Stoeter P. (2012). Missense *PANK2* Mutation without “Eye of the Tiger” Sign: MR Findings in a Large Group of Patients with Pantothenate Kinase-Associated Neurodegeneration (PKAN). J. Magn. Reson. Imaging.

